# A poisonous cocktail: interplay of cereulide toxin and its structural isomers in emetic *Bacillus cereus*

**DOI:** 10.3389/fcimb.2024.1337952

**Published:** 2024-03-26

**Authors:** Markus Kranzler, Veronika Walser, Timo D. Stark, Monika Ehling-Schulz

**Affiliations:** ^1^ Institute of Microbiology, Department of Pathobiology, University of Veterinary Medicine, Vienna, Austria; ^2^ Food Chemistry and Molecular and Sensory Science, Technical University of Munich, Freising, Germany

**Keywords:** cereulide isoforms, emetic toxin, food poisoning bacteria, *Bacillus cereus*, cytotoxicity, peptide toxin

## Abstract

Food intoxications evoked by emetic *Bacillus cereus* strains constitute a serious threat to public health, leading to emesis and severe organ failure. The emetic peptide toxin cereulide, assembled by the non-ribosomal peptide synthetase CesNRPS, cannot be eradicated from contaminated food by usual hygienic measures due to its molecular size and structural stability. Next to cereulide, diverse chemical variants have been described recently that are produced concurrently with cereulide by CesNRPS. However, the contribution of these isocereulides to the actual toxicity of emetic *B. cereus*, which produces a cocktail of these toxins in a certain ratio, is still elusive. Since cereulide isoforms have already been detected in food remnants from foodborne outbreaks, we aimed to gain insights into the composition of isocereulides and their impact on the overall toxicity of emetic *B. cereus*. The amounts and ratios of cereulide and isocereulides were determined in *B. cereus* grown under standard laboratory conditions and in a contaminated sample of fried rice balls responsible for one of the most severe food outbreaks caused by emetic *B. cereus* in recent years. The ratios of variants were determined as robust, produced either under laboratory or natural, food-poisoning conditions. Examination of their actual toxicity in human epithelial HEp2-cells revealed that isocereulides A-N, although accounting for only 10% of the total cereulide toxins, were responsible for about 40% of the total cytotoxicity. An this despite the fact that some of the isocereulides were less cytotoxic than cereulide when tested individually for cytotoxicity. To estimate the additive, synergistic or antagonistic effects of the single variants, each cereulide variant was mixed with cereulide in a 1:9 and 1:1 binary blend, respectively, and tested on human cells. The results showed additive and synergistic impacts of single variants, highlighting the importance of including not only cereulide but also the isocereulides in routine food and clinical diagnostics to achieve a realistic toxicity evaluation of emetic *B. cereus* in contaminated food as well as in patient samples linked to foodborne outbreaks. Since the individual isoforms confer different cell toxicity both alone and in association with cereulide, further investigations are needed to fully understand their cocktail effect.

## Introduction

The endospore-forming bacteria *Bacillus cereus* is a notorious food-borne pathogen that is gaining increasing prominence (Messelhäußer and Ehling-Schulz, 2018). In particular, emetic strains producing the depsipeptide toxin cereulide are known as an infamous source of food intoxications, with symptoms ranging from emesis and nausea to multi-organ failure and even death in some cases (Dierick et al., 2005; Naranjo et al., 2011; Tschiedel et al., 2015; Ehling-Schulz et al., 2019; Jovanovic et al., 2021; Thery et al., 2022). The ability of cereulide to cross the blood-brain barrier (BBB) (Bauer et al., 2018) may explain the cerebral effects, including acute encephalopathy, reported from severe intoxication cases (Ichikawa et al., 2010; Kubota et al., 2022). Cereulide is produced by the non-ribosomal peptide synthetase CesNRPS encoded by the *ces* genes on the megaplasmid pCer270, which shares its backbone with the *Bacillus anthracis* toxin plasmid pX01 (Ehling-Schulz et al., 2005b, Ehling-Schulz et al., 2006). Multiple realms of regulations ensure the tight control and right timing of cereulide toxin synthesis to embed it in the bacterial life cycle (Ehling-Schulz et al., 2015; Lücking et al., 2015; Dietrich et al., 2021). Although the *ces* genes are highly conserved in emetic *B. cereus*, the capacity for cereulide production is highly variable between the different strains (Stark et al., 2013). Generally, cereulide is pre-formed in foods contaminated with emetic *B. cereus*. Its chemical structure makes it resistant to heat treatment, proteolysis, and hydrolysis (Ehling-Schulz et al., 2004; Rajkovic et al., 2008). Furthermore, due to its small size (1.2 kDa), it cannot be removed by commonly used procedures in food processing, such as microfiltration and bactofugation.

In the dairy industry, for example, high-speed centrifugation, known as bactofugation, is used to reduce the number of bacterial cells and spores in food products (Ribeiro et al., 2019). While up to 90% of bacterial cells and spores can be removed from raw milk by this centrifugation process (Stack and Sillen, 1998), cereulide is retained. Thus, even hygienic measures allowing the inactivation and/or removal of vegetative cells and spores are ineffective against cereulide. Serious foodborne intoxications and fatalities result from the amount of toxin in contaminated food due to the versatile potential of cereulide production by emetic *B. cereus* strains, which depends on intrinsic (genetic) and extrinsic factors, such as the composition of food matrices, temperature, pH, and oxygen (Dommel et al., 2010; Stark et al., 2013; Messelhausser et al., 2014; Delbrassinne et al., 2015; Ehling-Schulz et al., 2015; Kranzler et al., 2016; Rouzeau-Szynalski et al., 2020). Lately, one of the highest concentrations of cereulide was detected in fried rice balls (37 µg cereulide/g foodstuff) responsible for an outbreak in Austria, with one patient suffering acute liver failure (Schreiber et al., 2022).

In recent years, various chemical cereulide variants, designated isocereulides, have been identified (Pitchayawasin et al., 2004; Marxen et al., 2015a, Marxen et al., 2015b; Walser et al., 2021, Walser et al., 2022), which are simultaneously produced in the same strain by the same CesNRPS (Ehling-Schulz et al., 2015; Marxen et al., 2015a; Walser et al., 2022). However, the amount and relative composition of the cereulide/isocereulide mixtures highly depend on extrinsic factors, such as temperature, substrate availability and food matrices (Marxen et al., 2015b; Kranzler et al., 2016). The CesNRPS is a multi-enzyme complex, recently shown to be tethered to the bacterial cell membrane by an ABC transporter (designated cesC/D) located next to the structural *ces* genes *cesA* and *cesB* in an operon (Dommel et al., 2010; Gacek-Matthews et al., 2020), produces cereulide via the non-ribosomal pathway (Ehling-Schulz et al., 2005b; Magarvey et al., 2006). The originally described cereulide with its dodecadepsipeptide structure is composed of alternating amino- and α-hydroxy acid modules, consisting of D- α -hydroxyisocapryl-D-alanyl-L- α-hydroxyisovaleryl-L-valyl (Agata et al., 1994). However, as substrate specificity of the CesNRPS appears not as stringent as previously thought, incorporation of different monomers on various positions by the CesNRPS is possible, leading to a variety of isocereulides. Moreover, also other amino- and hydroxy acids, such as serine (Ser), glycine (Gly), or 2-hydroxybutanoic acid (Abu), were found to be incorporated into the precursor subunits (Marxen et al., 2015a; Walser et al., 2022). Using a HEp2 cell-based cytotoxicity assay, it could be shown that the toxigenic potential of the newly identified isocereulides varies greatly. It ranges from approx. 8-fold higher cytotoxicity to less than 50% cytotoxicity compared to cereulide (Marxen et al., 2015a; Walser et al., 2022). Based on the results from structural chemical analyses and *in vitro* cytotoxicity studies of single isocereulides, we hypothesized that the actual toxicity of emetic *B. cereus* may not be due to cereulide exclusively, but rather to a combined additive or synergistic effect of cereulide together with its chemical structure variants. Thus, in this study, we aimed to gain insights into the composition of cereulide toxins, namely cereulide and isocereulides (= (iso)cereulide(s)), in bacteria grown under standard laboratory conditions and in food implicated in a severe foodborne outbreak. Furthermore, a comprehensive cytotoxicity study was carried out to decipher the biological function and contribution of isocereulides to the toxicity of emetic *B. cereus*.

## Material and methods

### Bacterial strains and growth conditions for iso(cereulide)s composition studies

To investigate the composition of cereulide and isocereulides (= iso(cereulide)s) in bacterial cultures grown under standard laboratory conditions, the emetic *B. cereus* reference strain F4810/72 (also termed AH187) was used to inoculate LB-Miller (LB) broth (10 g tryptone, 5 g yeast extract and 10 g NaCl per liter) with 10^3^ CFU/ml derived from a serial diluted overnight culture grown at 30°C in LB broth for 16 h. Cultures were grown in baffled flasks at 30°C with shaking (120 rpm) for 17, 19, 24, and 32 h, respectively. Bacterial cells were harvested by centrifugation (4 min, 8,000 *g*, room temperature) at indicated time points and stored at -20°C until further use. Cereulide and isocereulides were extracted from the bacterial cell pellets by shaking overnight at room temperature with 99.9% EtOH (high-performance liquid chromatography [HPLC] grade; AustrAlco), followed by centrifugation (12,000 *g*, 4 min) and filtration (0.2 µm), and analyzed by means of ultraperformance liquid chromatography-electrospray ionization-time of flight mass spectrometry (UPLC-ESI-TOF-MS) as described previously (Marxen et al., 2015a, Walser et al., 2022).

### Biofermentative generation and isolation of cereulide and isocereulides A–N

Cereulide and isocereulides were produced using a biotechnological approach described previously (Bauer et al., 2010). In order to obtain the amounts of purified cereulide and toxin variants needed for this study, the *B. cereus* reference strains F4810/72 and F4810/72/SCV/AN, a mutant strain showing accelerated cereulide production (Frenzel et al., 2015), were cultivated under standard laboratory conditions as described above for 24 h, and harvested by centrifugation (4 min, 8,000 *g*, room temperature). The supernatant was discarded, and pellets were autoclaved (121°C, 15 min). Subsequently, bacterial pellets were extracted with 99.9% EtOH as described above and the purification of the ethanolic extracts by solid-phase extraction, and semi-HPLC pre-fractionation was performed as described previously (Marxen et al., 2015a; Walser et al., 2021, Walser et al., 2022). The corresponding HPLC fractions were analyzed for cereulide and the known isocereulides A-N via UPLC-ESI-TOF-MS as described previously (Marxen et al., 2015b; Walser et al., 2022). Analytical purification was performed on an HPLC system equipped with a 250 × 4.6 mm, S-5 µm, YMC-Triart C18 column (YMC Europe, Dinslaken, Germany). The pre-fractionated sample was dissolved in ethanol and separated at a flow rate of 1 mL/min while monitoring the effluent at 220 nm using the gradients and parameters described in the literature (Walser et al., 2022).

### Quantitation of cereulide and the isocereulides by UPLC-ESI-MS/MS

For the quantitation of cereulide and the isocereulides A–N a previously established SIDA LC-MS/MS method was used (Bauer et al., 2010; Marxen et al., 2015b; Walser et al., 2022). In brief, all samples were spiked with the internal standard ^13^C_6_-cereulide (100 ng/mL) and 2 µL were injected into a Waters Xevo TQ-S mass spectrometer (Waters, Manchester, UK) combined with an Acquity UPLC i-class (Waters, Manchester, UK). Additionally, the system entailed a sample manager, binary solvent manager and column oven (Waters, Manchester, UK) and was operated with the software module MassLynx 4.1 SCN 813 while data processing was performed with the module TargetLynx. Chromatographic and mass spectrometric parameters were applied as described previously (Marxen et al., 2015b; Walser et al., 2022). To quantify cereulide and isocereulides A–N, the respective ammonium adducts were used during the multiple reaction monitoring mode and were chosen as follows: quantifier *m/z* 357.2; cereulide and isocereulide Gas *m/z* 1170.7 → qualifier *m/z* 172.2, 314.2; quantifier *m/z* 357.2;^13^C_6_-cereulide as *m/z* 1176.7 → qualifier *m/z* 173.2, 316.2; quantifier *m/z* 358.2; isocereulide D and J as *m/z* 1142.6 → qualifier *m/z* 172.2, 314.2; quantifier *m/z* 357.2; isocereulides B, E, H, I, and M as *m/z* 1156.6 → qualifier *m/z* 172.2, 314.2; isocereulide A, F and K as *m/z* 1184.6 → qualifier *m/z* 172.2, 314.2; quantifier *m/z* 357.2; and isocereulide C, L and N with *m/z* 1186.6 → qualifier *m/z* 172.2, 314.2; quantifier *m/z* 357.2. Based on calibration mixtures with cereulide (0.1–1000 ng/mL in EtOH) and the internal standard ^13^C_6_-cereulide (100 ng/mL), a calibration curve was created plotting the respective peak area ratios. All calibration points were analyzed in technical triplicates.

### Iso(cereulide)s cytotoxicity assay

To explore the role of isocereulides in the toxicity of emetic *B. cereus*, different cereulide toxin blends were prepared and subjected to a previously established HEp2 bioassay (Lücking et al., 2009). To obtain blends with the desired toxin concentrations, appropriate volumes of cereulide and single iCer fractions were mixed and applied to HEp-2 cells. Next to the toxin blends, pure cereulide and single iCer toxins were tested in the HEp-2 bioassay for reasons of comparison.

In brief, the toxin fractions to be assayed were serially two-fold diluted in MEM-Earle medium (Sigma-Aldrich), containing 2% ethanol, 2% FCS (v/v; Merck), 1% sodium pyruvate (Pan), and 0.4% penicillin‐streptomycin (v/v; Sigma-Aldrich), in 96-well microtiter plates (Greiner Bio-One, Austria). HEp-2 cells (10^5^ cells/well) were added to the wells and the plates were incubated for 48 h at 37°C in a 5% CO_2_ atmosphere. Cell viability was determined spectrophotometrically using the CCK8 reagent (Bimake) (10 µl/well). Pure cereulide served as a control. To investigate the relative cytotoxicity, the half-maximal effective concentration (EC_50_) of cereulide toxins and toxin blends were determined and the reciprocal EC_50_-values (1/EC_50_) were calculated. Cytotoxicity of isocereulides and blends was calculated relative to cereulide (EC_50_ = 2.44 (+/-0.27) ng/ml), which was set to =1. Statistically significant differences (p < 0.05; 0.01; 0.001) in cytotoxicity of isocereulides and blends compared with cereulide were calculated using a two-sided t-test.

The effect of a binary blends was designated as ‘synergistic’ when a combined effect of a binary blend containing cereulide plus one isocereulide was greater than the expected sum of the separate effects of cereulide plus the respective isolcereulide. An effect of a binary blend was designated as ‘antergonistic’ when a combined effect of a binary blend containing cereulide plus one isocereulide was lower than the expected sum of the separate effects of cereulide plus the respective isolcereulide.

## Results and discussion

While cereulide, the emetic toxin produced by a certain group of *B. cereus* (Ehling-Schulz et al., 2005a), was identified and characterized about 30 years ago (Agata et al., 1994, Agata et al., 1995) much less is known about the recently identified cereulide variants, the so-called isocereulides (Marxen et al., 2015a; Walser et al., 2022). Therefore, we analyzed the composition of isocereulides in bacterial cultures grown under laboratory standard conditions as well as in food remnants from an outbreak and investigated their potential contribution to the toxicity of emetic *B. cereus*. Since interference of compounds from food matrices may impact the quantitation of molecules, such as peptide toxins or pesticides, in foods (Song et al., 2019), we used a previously established stable isotope dilution assay (SIDA) to correct for matrix effects (Bauer et al., 2010). This SIDA, which formed the basis for the establishment of the ISO18465:2017, has been successfully employed for quantitation of cereulide and isocereulides in foods linked to emetic outbreaks and patient specimen (Marxen et al., 2015b; Schreiber et al., 2022).

### Biofermentative production and purifications of iso(cereulide)s

First, a previously developed biotechnological approach to generate cereulide in large quantities and high purity (Bauer et al., 2010) was used to produce cereulide and the isocereulides A-N (iCerA - iCerN) by biofermentation. For this purpose, the emetic reference strain F4810/72 and an isogenic mutant with accelerated cereulide synthesis (Frenzel et al., 2015) were grown under standard laboratory conditions, and cereulide together with the isocereulides was extracted from the pelleted bacterial cells with ethanol as described in the material and method section. After prefractionation of the extracts by RP18 solid-phase extraction, purified fractions of cereulide and isocereulides A-N were obtained by means of preparative HPLC followed by analytical HPLC purification. This procedure allowed us to generate cereulide and isocereulides in large amounts and high purity, with only minor traces of other substances. For cereulide and iCerG a purity of >99% was achieved while the purity of iCerB, iCerC, iCerD, iCerH, iCerI, iCerJ, iCerK and iCerM ranged between 93% and 99% (**Table 1**). However, due to the similar m/z ratios and hydrophobic properties of the cereulide toxins (Marxen et al., 2015a; Walser et al., 2022), we were not able to separate the isocereulides F/A, and L/N, respectively. Thus, these isocereulides were assayed as mixtures. The iCerF fraction consisted of 63% iCerF and 37% iCerA while the determination of the single ratios was not possible for the L/N fraction. As the total percentage of iCer L/N was determined to be 100% in this binary fraction, we included the latter as a mixture, designated iCerL/N (**Table 1**).

**Table 1:** Biofermentative production and isolation of iso(cereulide)s.

Toxin fractions	Relative amount of toxin per fraction (%)														
	*Cer*	*iCer A*	*iCer B*	*iCer C*	*iCer D*	*iCer E*	*iCer F*	*iCer G*	*iCer H*	*iCer I*	*iCer J*	*iCer K*	*iCer L/N*	*iCer M*	*total*
***Cer***	**99.23**	0.05	0.00	0.00	0.00	0.00	0.00	0.64	0.00	0.00	0.01	0.07	0.00	0.00	100.00
***iCer A***	0.63	**98.70**	0.00	0.00	0.00	0.00	0.00	0.45	0.00	0.00	0.00	0.22	0.00	0.00	100.00
***iCer B***	0.34	0.00	**92.83**	0.00	0.00	5.77	0.00	0.00	0.00	0.00	1.06	0.00	0.00	0.00	100.00
***iCer C***	2.24	0.00	0.00	**97.76**	0.00	0.00	0.00	0.00	0.00	0.00	0.00	0.00	0.00	0.00	100.00
***iCer D***	0.11	0.00	0.00	0.94	**96.43**	0.00	0.00	0.00	2.53	0.00	0.00	0.00	0.00	0.00	100.00
***iCer F***	0.00	37.47	0.00	0.00	0.00	0.00	**62.53**	0.00	0.00	0.00	0.00	0.00	0.00	0.00	100.00
***iCer G***	0.00	0.73	0.00	0.00	0.00	0.00	0.00	**99.07**	0.00	0.00	0.00	0.20	0.00	0.00	100.00
***iCer H***	8.05	0.00	0.00	0.14	0.16	0.00	0.00	0.00	**88.81**	1.35	1.49	0.00	0.00	0.00	100.00
***iCer I***	8.40	0.00	0.00	0.00	0.03	0.00	0.00	0.07	0.00	**59.08**	32.43	0.00	0.00	0.00	100.00
***iCer J***	11.10	0.00	0.00	0.00	0.00	0.00	0.00	2.69	0.00	0.99	**85.09**	0.13	0.00	0.00	100.00
***iCer K***	0.57	2.83	0.00	0.00	0.00	0.00	0.00	3.46	0.00	0.00	0.00	**93.13**	0.00	0.00	100.00
***iCer L/N***	0.00	0.00	0.00	0.00	0.00	0.00	0.00	0.00	0.00	0.00	0.00	0.00	**100.00**	0.00	100.00
***iCer M***	6.83	0.00	0.00	0.00	0.00	0.00	0.00	0.00	0.00	0.00	0.00	0.00	0.00	**93.17**	100.00

^a)^Cereulide and its chemical cereulide variants, designated isocereulides, were produced by biofermentation using a previously described biotechnological approach (Bauer et al., 2010). After prefractionation of the extracts by RP18 solid-phase extraction, purified fractions of cereulide and isocereulides A-N were obtained by means of preparative HPLC followed by analytical HPLC purification. The relative amounts (%) of cereulide and its variants in the purified fraction are depicted.The major components of the toxin fractions are printed in bold face.

### Composition of iso(cereulide)s produced by *B. cereus* under standard laboratory conditions

**Figure 1:**
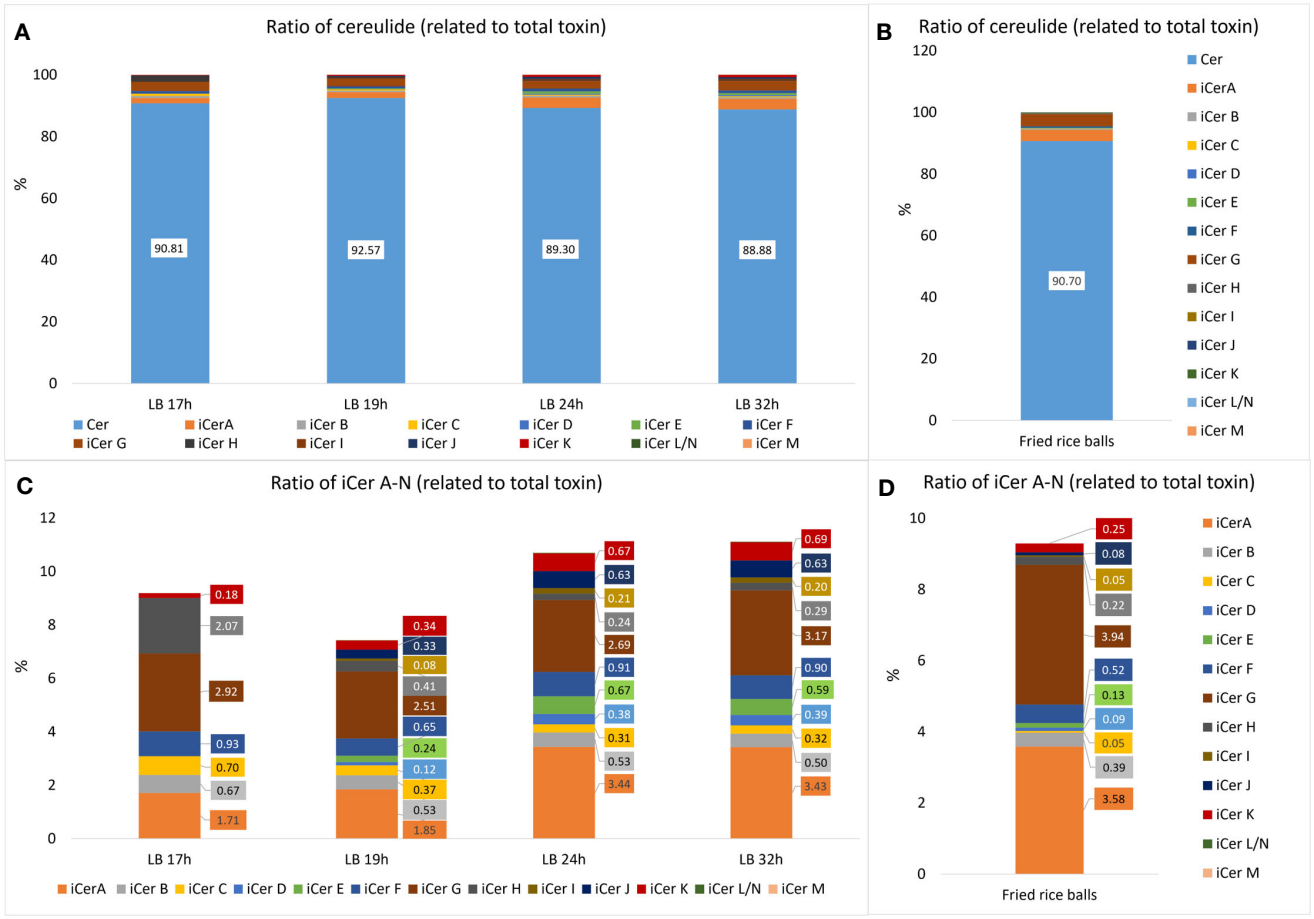
Relative amounts of cereulide (Cer) and isocereulides A-N (iCerA-N) produced by the emetic *B*. *cereus* reference strain F4810/72 (AH187) grown under laboratory standard conditions (30°C, LB broth, 120 rpm shaking) compared to the relative amounts found in food implicate in a foodborne outbreak (Schreiber et al., 2022). **(A)** Overall ratios of cereulide (Cer) and single cereulide variants iCer A to iCerN (iCerA-N) calculated relative to the total cereulide toxins (Cer+iCerA-N). **(B)** Relative amounts of cereulide and iCerA-N found in fried rice balls implicated in a foodborne outbreak. **(C)** Zoom into A, depicting the ratios of single cereulide variants iCer A to iCerN calculated relative to the total cereulide toxins. Samples for cereulide and isocereulide analysis by UPLC-MS/MS were taken after 17, 19, 24 and 32 hours respectively to follow the dynamics of cereulide and isocereulide production. **(D)** Zoom into B, depicting the ratios of single cereulide variants iCerA-N found in fried rice balls implicated in a foodborne outbreak.

Since we showed previously that cereulide accumulates in the stationary phase (Dommel et al., 2011; Kalbhenn et al., 2022) we analyzed the composition of cereulide and its isoforms, designated as iso(cereulide)s, after growth in standard laboratory conditions (LB, 30°C, 120 rpm; for details see material and methods) for 17h, 19h, 24h, and 32h, respectively. As shown in **Table 2**, the total amount of cereulide toxins increased significantly between 17h and 24h. The overall ratio cereulide to isocereulides A-N was found to be approx. 9:1, independent of the total amount of iso(cereulide)s at the different time points of toxin quantitation by UPLC-MS/MS. The range of the iCer A-N ratio compared to the total toxin amount varied between 7.4% (19 hours) and 11.1% (32 hours), with an average of 9.6% (+/- 1.45) (**Figure 1A**).

**Table 2:** Quantitation of cereulide and its variants by UPLC-ESI-MS/MS.

Time	µg toxin per g bacterial wet weight														
	*Cer*	*iCerA*	*iCer B*	*iCer C*	*iCer D*	*iCer E*	*iCer F*	*iCer G*	*iCer H*	*iCer I*	*iCer J*	*iCer K*	*iCer L/N*	*iCer M*	*total*
***17h***	8.26	0.16	0.06	0.06	0.00	0.00	0.08	0.27	0.19	0.00	0.00	0.02	0.00	0.00	9.10
***19h***	123.90	2.47	0.70	0.50	0.16	0.32	0.87	3.35	0.54	0.11	0.45	0.45	0.01	0.01	133.85
***24h***	580.64	22.36	3.47	2.00	2.50	4.34	5.91	17.50	1.55	1.35	4.11	4.35	0.08	0.06	650.24
***32h***	377.08	14.55	2.10	1.36	1.64	2.51	3.82	13.44	1.21	0.85	2.69	2.91	0.06	0.05	424.27

^a)^The emetic B. cereus reference strain F4810/72 was grown under laboratory standard conditions (Frenzel et al., 2012). Samples for toxin quantitation were taken at the indicated time points. Cells were harvested by centrifugation and cereulide was extracted from the bacterial pellets along with its variants using 99.9% ethanol. Quantitation of cereulide and isocereulides was carried out using a SIDA LC-MS/MS method. Toxin amounts are given as µg cereulide toxins/g bacterial wet weight.

Notably, the analysis of single cereulide variants revealed differences in the relative composition of isocereulides after distinct culture times. Thus, integrated metabolome studies, as recently described for *Lactobacillus* (Le et al., 2023), after different culture times might be interested to elucidate potential links between the isocereulide composition and the bacterial metabolome in future studies. The ratios between isocereulides were found to be comparable at 24h and 32h but were apparently different at 17h and 19h (**Figure 1B**). The most abundant variant at 17h was iCerG (3.2%), followed by iCerH (2.3%). The levels of iCerG remained high (between 2.5 and 3.2%) over the time course of measurements while the relative amount of iCerH declined to 0.4% after 19h, and to 0.3% after 24h and 32h, respectively. In contrast, iCer A accounted for 1.9% and 2.0% at 17h and 19h, respectively, but was the most abundant isocereulide (3.4%) at 24h and 32h. Similarly, to iCerH, the highest relative amount of iCerC was found at 17h (0.7%) whilst it only accounted for 0.4% after 19h and slightly declined thereafter to 0.3%. The fourth most abundant isocereulides iCerF (0.9%) was found in similar relative amounts over the complete time course of measurements while the fifth most abundant isocereulide iCerB at 17h slightly declined from 0.7% at 17h to 0.5% at 32h. iCerK, which indicated an opposite trend, increased steadily from 0.2% at 17h to 0.7% at 32h. Finally, iCerD, iCerE, iCerI and iCerJ, which were too low to be determined at 17h, also showed increasing concentrations from 19h onwards but remained below 1% even at 24h and 32h (**Figure 1B**). iCerN/L and iCerM were not detectable at 17h and thereafter only in traces. Overall, our results point towards different kinetics of isocereulide production, which may reflect the preference of CesNRPS for the activation and uptake of specific substrates (Magarvey et al., 2006) and/or may result from the availability of certain amino acids and hydroxy acids at distinctive time points.

### Composition of iso(cereulide) in contaminated food linked to a severe outbreak

Next, we investigated the isocereulide composition of fried rice balls implicated in a recent foodborne outbreak (Schreiber et al., 2022), to gain insight into the potential role of isocereulides in foodborne intoxication. Analyses of food remnants from the rice balls by UPLC-ESI-MS/MS (Marxen et al., 2015a; Walser et al., 2022) revealed that the original cereulide accounts for 90.7% while isocereulides A-N account for 9.3%, of the total amount of cereulide toxins (**Figure 1C**). These data are in line with the results from the analysis of iso(cereulide)s composition of the emetic reference strain F4810/72 under standard laboratory conditions, with an average ratio for isocereulides A-N of 9.6% (+/- 1.45%). Thus, it could be expected that isocereulides constitute approx. 10% of the total toxin amount under laboratory culture conditions as well as in foods implicated in food poisoning. However, further studies will be necessary to elucidate if the observations from our current work can be generalized. Similarly, to the experiments under standard laboratory conditions, iCerA and iCerG were the most prevalent isocereulides found in the contaminated rice balls (**Figure 1D**). Notably, iCerA and iCerG are the two isocereulides previously detected in trace amounts in the serum and the urine of the patient suffering from acute liver failure after consumption of the contaminated rice balls (Schreiber et al., 2022). These results support the hypothesis that isocereulides may indeed play a role in food poisoning caused by *B. cereus* which remains to be explored and underscore the need to fully understand their biological function. iCerA constitutes 3.6% and iCerG 3.9% of total cereulide toxins in the contaminated rice balls, which might explain why these two isocereulides but not the other isocereulides have been detectable by UPLC-MS/MS analyses in the patient samples. iCerF accounts for 0.5%, iCerB for 0.4%, iCer K for 0.3%, iCerH for 0.2% and iCerE for 0.1%. All other isocereulides (except iCerL/N and iCerM which were not detectable at all) were present in amounts below 0.1% and found to be generally lower in the rice balls (iCerC 0.05%, iCerD 0.09%, iCerI 0.05% and iCerJ 0.08%), than in the emetic reference strain cultivated under laboratory conditions.

### Cytotoxic effects of cereulide and iCer A-N in natural ratios

A bioassay based on human HEp-2cells, previously established to evaluate the toxicity of cereulide (Finlay et al., 1999; Lücking et al., 2009; Frenzel et al., 2012), was employed to investigate the cytotoxic effect of cereulide and its chemical variants in their naturally formed ratio by *B. cereus*. In order to elucidate the contribution of isocereulides to the overall cytotoxicity, different mixtures of cereulide toxins were prepared to mimic the natural composition of the iso(cereulide)s at different time points of *B. cereus* grown under standard laboratory conditions as well as the natural composition of iso(cereulide)s observed in fried rice balls implicated in a recent foodborne outbreak. These mixes of cereulide toxins were designated as ‘Cer+iCerA-N’. In addition, blends, designated as iCerA-N, were prepared that only contained the isocereulides A-N but were lacking the original cereulide. To obtain blends with the desired concentrations, appropriate volumes of the single iCer fractions and cereulide were mixed and applied to HEp-2 cells. All blends were normalized to the corresponding fractions containing pure cereulide, which were set to 1.

**Figure 2:**
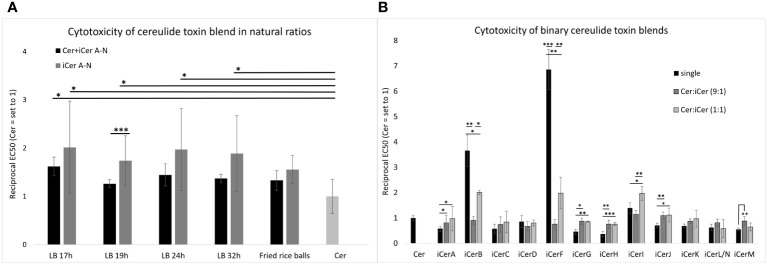
*In vitro* cytotoxicity of cereulide, isocereulides and binary cereulide + isocereulide cocktails towards HEp-2 cells. The EC_50_ values were determined to investigate the cytotoxicity of the single cereulide toxins as well as the cytotoxicity of different toxin blends, towards HEp-2 cells. The EC_50_ value referrers to the concentration of the toxins/toxin blends at which 50% cytotoxicity towards HEp-2 cells was observed. The reciprocal EC_50_values are shown for clarity. Higher reciprocal EC_50_values indicate higher cytotoxicity. **(A)** Cytotoxic effect of total cereulide toxins (Cer + iCer A-N) and isocereulide (iCer A-N) toxin blends mixed in natural ratios (for details see **Figure 1**). **(B)** Cytotoxic effect of cereulide and isocereulide A-N in single fractions, and binary blends of cereulide and single isocereulides (Cer:iCer) 9:1 and 1:1 blends, respectively. Singular values of iCer H-N were adopted from Walser et al., 2022. Cytotoxicity titers of single isocereulides and toxin blends were calculated relative to the cytotoxicity titer of pure cereulide (EC_50 = _2.44 (+/-0.27) ng/ml), which was set to 1. Error bars represent standard deviations from the mean values derived from three independent biological replicates. Statistically significant differences in cytotoxicity titers of toxin blends compared with the cereulide titer **(A)** or the respective single isocereulide titers **(B)** were calculated with a paired, two-tailed Student’s t-test and are marked with asterisks as follows: * p<0.05, ** p<0.01, *** p<0.001.

The cytotoxic potential (reflected by the reciprocal EC_50_ values when referenced to pure cereulide) of cereulide mixed with isocereulides A-N (designated as Cer+iCerA-N) at natural ratios was enhanced compared to pure cereulide. Cytotoxicity of the respective Cer+iCerA-N blends ranged from 1.3-fold to 1.6-fold compared to that of pure cereulide (**Figure 2A**), indicating that the 90% amount of cereulide may contribute to 60% of the estimated toxicity, whereas the remaining 10% of isocereulides may account to approximately 40%. To test this hypothesis that isocereulides A-N cocktails, consisting of isocereulides A-N in natural proportions, account for higher cytotoxicity than pure cereulide, we examined the effect of isocereulides A-N mixtures without cereulide (=iCerA-N). Indeed, the toxicity towards Hep2-cells of iCerA-N was found to be significantly higher than that of pure cereulide and even higher than that of blends containing cereulide and isocereulides A-N mixtures (=Cer+iCerA-N). The respective iCerA-N blends, which mimic the natural ratios of isocereulides in bacteria grown under laboratory standard conditions (Frenzel et al., 2012), showed about 1.6- to 1.9-fold cytotoxicity compared to pure cereulide. Similarly, the iCerA-N blends, which mimicked the natural ratio of isocereulides found in fried rice balls that led to severe food poisoning (Schreiber et al., 2022), also showed 1.5-fold higher cytotoxicity than cereulide (see **Figure 2A**). Thus, it can be concluded that the toxicity of isocereulides A-N in natural ratios is about 70% enhanced compared to pure cereulide. These results highlight the importance of unraveling the biological and ecological function of these cereulide isoforms.

### Insights into potential additive, synergistic, or antagonistic effects of iso(cereulide)s

To explore a potential synergistic, additive, or antagonistic mode of action of cereulide and specific isocereulides, binary blends containing cereulide with each particular isocereulide were prepared and subjected to the HEp-2 bioassay. First, we tested blends at a 9:1 ratio of cereulide with each iCer to elucidate whether any of the isocereulides would be dominant in efficacy and contribution to cytotoxicity. Some isocereulides, namely iCerB (3.5-fold), iCerF (7-fold) and iCerI (1.4-fold), showed higher cytotoxicity than the original cereulide when tested as single components (**Figure 2B**), see also (Walser et al., 2022), suggesting that these isocereulides play a pivotal role in the enhanced cytotoxicity observed in blends with natural ratios of iCer A-N compared to pure cereulide (**Figure 2A**). However, our results revealed no significant difference between pure cereulide and toxin blends with 9:1 ratio of cereulide and each iCer, indicating that the poising potential of emetic *B. cereus* rather relates to the cocktail effect of iso(cereulide)s than to a single toxin variant (**Figure 2B**).

Next, we tested the cytotoxicity of blends at a 1:1 ratio of cereulide with each iCer to gain insights into potential interplays between cereulide and iCer. As depicted in **Figure 2B**, the highly toxic cereulide variants iCerB and iCerF showed significantly higher cytotoxicity in the 1:1 mixture with cereulide than in the 9:1 mixture, but significantly lower cytotoxicity than the respective pure iCer, suggesting additive effects between cereulide and these highly toxic isocereulides. In contrast, the 1:1 mixture of cereulide and iCerI showed significantly higher cytotoxicity than the single cereulide toxins or the 9:1 blend, demonstrating a synergistic effect of cereulide and iCerI. Cereulide variants with lower toxicity than cereulide revealed a versatile efficacy in the 1:1 binary blends. A synergistic effect, although not as pronounced as between cereulide and iCerI, was observed between cereulide and the following isocereulides: iCerA, iCerG, iCerH, and iCerJ. These isoforms showed a similar or slightly higher cytotoxicity in the 1:1 mixture than in the 9:1 although we theoretically expected a lower cytotoxic effect for the 1:1 blends as it was observed for the 1:1 mixture of cereulide and iCerM. These results may reflect enhanced toxicity of these isoforms when combined with cereulide. Notably, the 9:1 ratio mixture of cereulide and iCerD showed a slightly lower cytotoxicity than pure iCerD or the 1:1 mixture, although iCerD alone is less cytotoxic than cereulide. The latter results may be indicative of a possible antagonistic effect between cereulide and iCerD (**Figure 2B**). It is still elusive how the toxicity of isocereulides is elicited but it may be plausible that altered traits in potassium scavenging and hydrophobicity (Marxen et al., 2015a) lead to altered host cell sensitivity to different combinations of cereulide toxins. While the *in vitro* cytotoxicity results represented here are an important first step in characterizing the toxicity of the isocereulide variants and their ability to synergize with cereulide, future *in vivo* animal studies will be needed to validate these results and evaluate *in vivo* toxicity and serum concentrations. In summary, our results indicate complex interactions between the distinct cereulide toxins, which warrants further studies to fully understand the biological function of these highly stable cyclic depsipeptides. Such studies would be important for the development of a knowledge-based risk assessment of emetic *B. cereu*s.

## Conclusion

This study provides first insights into the interaction of cereulide and variants. It emphasises the importance of the cereulide variants for the overall toxicity of the emetic *B. cereus*. Our work revealed that the originally described cereulide, which constitutes 90% of total toxin, accounts for only 60% of the toxicity of cereulide toxins while iCer A-N, which constitutes only 10% of total toxin, accounts for 40% of toxicity. Furthermore, we demonstrated that toxicity is not due to one specific, highly cytotoxic iCer next to cereulide, but rather results from the complex interplay (including synergistic, additive, and antagonistic actions) between cereulide and its variants. Notably, the mix of cereulide and iCerA-N produced under natural conditions was found to be a toxic cocktail. Thus, it would be of utmost importance to include not only cereulide but also isocereulides in routine food microbiology and clinical diagnostics in order to obtain a valid assessment of the toxicity of emetic *B. cereus* and to be able to take evidence-based measures in outbreak situations. To this end, recent developments in the application of metabolomics for food safety control, which allows the detection of more than 1000 molecules in food (including contaminants), could contribute to the introduction of an integrated system for the automatic detection of cereulide and isocereulide in the near future (Li et al., 2021).

## Data availability statement

The original contributions presented in the study are included in the article/supplementary material. Further inquiries can be directed to the corresponding author.

## Ethics statement

Ethical approval was not required for the studies on humans in accordance with the local legislation and institutional requirements because only commercially available established cell lines were used.

## Author contributions

MK: Writing – original draft, Writing – review & editing, Data curation, Formal analysis, Investigation, Methodology, Visualization. VW: Writing – original draft, Data curation, Formal analysis, Investigation, Methodology. TS: Writing – review & editing, Conceptualization, Funding acquisition, Project administration, Supervision. ME-S: Conceptualization, Funding acquisition, Project administration, Supervision, Writing – review & editing, Formal analysis, Resources, Writing – original draft.
